# Supplementary material for: Comparison of Risk Factors for Cholangiocarcinoma and Hepatocellular Carcinoma: A Prospective Cohort Study in Korean Adults
Source: Cancers (Basel). 2022 Mar 28;14(7):1709. doi: 10.3390/cancers14071709 (PMC8997058; doi:10.3390/cancers14071709)
Supplement: Supplementary file 1 [file cancers-14-01709-s001.zip › cancers-1614374-supplementary.pdf]

Supplementary Materials: Comparison of Risk Factors for Cholangiocarcinoma and Hepatocellular Carcinoma: A Prospective Cohort Study in Korean Adults

In Rae Cho, Sang–Wook Yi, Ja Sung Choi and Jee–Jeon Yi

Table S1. HRs for CCA, iCCA, eCCA, and HCC after adjustment of Sex and Age.

Variable/Group	CCA (*n* = 1388)			iCCA (*n* = 821)			eCCA (*n* = 567)			HCC (*n* = 2920)		
	p	HR	(95%CI)	*p*	HR	(95%CI)	*p*	HR	(95%CI)	*p*	HR	(95%CI)
Age, years												
Per 10–year older	<0.001	2.55	(2.10–3.09)	<0.001	2.29	(1.79–2.94)	<0.001	2.99	(2.21–4.04)	<0.001	1.61	(1.41–1.84)
Sex, men (vs. women)	<0.001	2.24	(2.00–2.51)	<0.001	2.28	(1.97–2.65)	<0.001	2.18	(1.83–2.59)	<0.001	4.35	(3.95–4.78)
Smoking status												
Never smoker		1.00	(Reference)		1.00	(Reference)		1.00	(Reference)		1.00	(Reference)
Past smoker	0.111	1.17	(0.97–1.41)	0.297	1.14	(0.89–1.46)	0.216	1.20	(0.90–1.60)	0.354	1.06	(0.94–1.20)
Current smoker, <1 pack/day	<0.001	1.35	(1.17–1.56)	0.001	1.38	(1.14–1.66)	0.016	1.31	(1.05–1.64)	<0.001	1.31	(1.20–1.44)
≥1 pack/day	<0.001	1.62	(1.28–2.05)	<0.001	1.83	(1.37–2.44)	0.187	1.31	(0.88–1.97)	0.210	0.90	(0.76–1.06)
Alcohol use, g ethanol/day												
None		1.00	(Reference)		1.00	(Reference)		1.00	(Reference)		1.00	(Reference)
<10	0.352	0.93	(0.79–1.09)	0.176	0.87	(0.70–1.07)	0.858	1.02	(0.80–1.30)	<0.001	0.78	(0.71–0.87)
10–39	0.004	1.25	(1.08–1.46)	0.014	1.28	(1.05–1.55)	0.118	1.21	(0.95–1.55)	0.004	0.86	(0.78–0.95)
≥ 40	<0.001	1.69	(1.37–2.07)	<0.001	1.63	(1.25–2.13)	<0.001	1.77	(1.29–2.43)	<0.001	1.31	(1.14–1.49)
Body mass index, kg/m^2												
<18.5	0.234	0.81	(0.58–1.14)	0.190	0.73	(0.46–1.17)	0.763	0.93	(0.57–1.51)	0.980	1.00	(0.79–1.27)
18.5–24.9		1.00	(Reference)		1.00	(Reference)		1.00	(Reference)		1.00	(Reference)
25–29.9	0.176	1.08	(0.97–1.21)	0.364	1.07	(0.92–1.24)	0.306	1.10	(0.92–1.31)	0.321	0.96	(0.89–1.04)
≥30	0.016	1.43	(1.07–1.92)	0.135	1.34	(0.91–1.98)	0.050	1.56	(1.00–2.42)	0.211	1.15	(0.92–1.44)
Per 5kg/m^2 increase	0.008	1.12	(1.03–1.22)	0.124	1.09	(0.98–1.22)	0.023	1.17	(1.02–1.33)	0.531	0.98	(0.92–1.04)
Diabetes status (serum glucose, mg/dL)												
Normoglycemia (<100)		1.00	(Reference)		1.00	(Reference)		1.00	(Reference)		1.00	(Reference)
IFG (101–125)	0.222	1.08	(0.95–1.23)	0.891	0.99	(0.84–1.17)	0.036	1.23	(1.01–1.50)	0.039	1.10	(1.00–1.20)
Diabetes (≥126 or known diabetes)	<0.001	1.39	(1.21–1.61)	0.030	1.24	(1.02–1.50)	<0.001	1.64	(1.32–2.03)	<0.001	1.84	(1.67–2.02)
Per 18mg/dL increase	0.005	1.05	(1.02–1.09)	0.100	1.04	(0.99–1.10)	0.017	1.07	(1.01–1.13)	<0.001	1.12	(1.10–1.15)
Alanine aminotransferase (ALT), IU/L												
<20	<0.001	0.77	(0.68–0.87)	0.014	0.82	(0.71–0.96)	<0.001	0.70	(0.58–0.84)	<0.001	0.36	(0.32–0.41)
20–39		1.00	(Reference)		1.00	(Reference)		1.00	(Reference)		1.00	(Reference)
40–59	0.054	1.20	(1.00–1.44)	0.024	1.31	(1.04–1.65)	0.760	1.05	(0.78–1.41)	<0.001	3.60	(3.27–3.96)
60–79	0.014	1.47	(1.08–2.00)	0.012	1.64	(1.12–2.41)	0.420	1.24	(0.74–2.08)	<0.001	7.14	(6.34–8.05)
≥80	0.026	1.48	(1.05–2.09)	0.023	1.64	(1.07–2.53)	0.442	1.25	(0.70–2.23)	<0.001	12.47	(11.20–13.9)
Total cholesterol, mg/dL												
Per 39mg/dL increase	0.003	0.92	(0.87–0.97)	<0.001	0.86	(0.80–0.93)	0.912	1.00	(0.93–1.09)	<0.001	0.51	(0.49–0.53)
Comorbid liver disease												
Hepatitis B virus infection	<0.001	2.65	(1.56–4.49)	0.002	2.80	(1.45–5.41)	0.050	2.41	(1.00–5.83)	<0.001	22.03	(19.5–24.9)
Hepatitis C virus infection	0.272	1.89	(0.61–5.85)	0.283	2.14	(0.53–8.54)	0.669	1.53	(0.22–10.91)	<0.001	18.38	(14.1–23.9)
Liver cirrhosis	0.458	1.45	(0.54–3.87)	0.295	1.83	(0.59–5.69)	0.910	0.89	(0.13–6.36)	<0.001	44.50	(39.1–50.6)
Alcoholic liver disease	0.045	1.69	(1.01–2.81)	0.486	1.30	(0.62–2.75)	0.022	2.26	(1.12–4.56)	<0.001	3.63	(2.91–4.53)
Biliary tract disease												
Cholelithiasis (Gallstone disease)	<0.001	5.68	(3.56–9.04)	<0.001	4.90	(2.54–9.45)	<0.001	6.74	(3.49–13.0)	0.002	2.36	(1.37–4.07)

Choledocholithiasis (Bile duct stone)	<0.001	13.69	(7.56–24.79)	<0.001	10.92	(4.53–26.33)	<0.001	17.36	(7.76–38.8)	0.538	1.55	(0.39–6.19)

Abbreviations: HR, hazard ratio; CCA, cholangiocarcinoma; iCCA, intrahepatic cholangiocarcinoma; eCCA, extrahepatic cholangiocarcinoma; HCC, hepatocellular carcinoma; 95% CI, 95% confidence interval; IFG, impaired fasting glucose.
